# Tracking key virulence loci encoding aerobactin and salmochelin siderophore synthesis in *Klebsiella pneumoniae*

**DOI:** 10.1186/s13073-018-0587-5

**Published:** 2018-10-29

**Authors:** Margaret M. C. Lam, Kelly L. Wyres, Louise M. Judd, Ryan R. Wick, Adam Jenney, Sylvain Brisse, Kathryn E. Holt

**Affiliations:** 1 Department of Biochemistry and Molecular Biology, Bio21 Molecular Science and Biotechnology Institute, University of Melbourne, Parkville, Victoria 3010 Australia; 2 Biodiversity and Epidemiology of Bacterial Pathogens, Institut Pasteur, 75015 Paris, France; 3 Department of Infectious Diseases and Microbiology Unit, The Alfred Hospital, Melbourne, Victoria 3004 Australia; 4 London School of Hygiene & Tropical Medicine, London, WC1E 7HT UK

**Keywords:** *Klebsiella pneumoniae*, Virulence, Hypervirulence, Salmochelin, Aerobactin, Virulence plasmids, Plasmids, Invasive disease, Genomic surveillance

## Abstract

**Background:**

*Klebsiella pneumoniae* is a recognised agent of multidrug-resistant (MDR) healthcare-associated infections; however, individual strains vary in their virulence potential due to the presence of mobile accessory genes. In particular, gene clusters encoding the biosynthesis of siderophores aerobactin (*iuc*) and salmochelin (*iro*) are associated with invasive disease and are common amongst hypervirulent *K*. *pneumoniae* clones that cause severe community-associated infections such as liver abscess and pneumonia. Concerningly, *iuc* has also been reported in MDR strains in the hospital setting, where it was associated with increased mortality, highlighting the need to understand, detect and track the mobility of these virulence loci in the *K*. *pneumoniae* population.

**Methods:**

Here, we examined the genetic diversity, distribution and mobilisation of *iuc* and *iro* loci amongst 2503 *K*. *pneumoniae* genomes using comparative genomics approaches and developed tools for tracking them via genomic surveillance.

**Results:**

*Iro* and *iuc* were detected at low prevalence (< 10%). Considerable genetic diversity was observed, resolving into five *iro* and six *iuc* lineages that show distinct patterns of mobilisation and dissemination in the *K*. *pneumoniae* population. The major burden of *iuc* and *iro* amongst the genomes analysed was due to two linked lineages (*iuc1*/*iro1* 74% and *iuc2*/*iro2* 14%), each carried by a distinct non-self-transmissible IncFIB_K_ virulence plasmid type that we designate KpVP-1 and KpVP-2. These dominant types also carry hypermucoidy (*rmpA*) determinants and include all previously described virulence plasmids of *K*. *pneumoniae*. The other *iuc* and *iro* lineages were associated with diverse plasmids, including some carrying IncFII conjugative transfer regions and some imported from *Escherichia coli*; the exceptions were *iro3* (mobilised by ICE*Kp1*) and *iuc4* (fixed in the chromosome of *K*. *pneumoniae* subspecies *rhinoscleromatis*). *Iro*/*iuc* mobile genetic elements (MGEs) appear to be stably maintained at high frequency within known hypervirulent strains (ST23, ST86, etc.) but were also detected at low prevalence in others such as MDR strain ST258.

**Conclusions:**

*Iuc* and *iro* are mobilised in *K*. *pneumoniae* via a limited number of MGEs. This study provides a framework for identifying and tracking these important virulence loci, which will be important for genomic surveillance efforts including monitoring for the emergence of hypervirulent MDR *K*. *pneumoniae* strains.

**Electronic supplementary material:**

The online version of this article (10.1186/s13073-018-0587-5) contains supplementary material, which is available to authorized users.

## Background

The enteric opportunistic bacterial pathogen *Klebsiella pneumoniae* imposes an increasing infection burden worldwide [1, 2]. These infections typically fall into one of two distinct categories: healthcare-associated (HA) infections caused by strains that are frequently multidrug-resistant (MDR) and community-associated (CA) infections arising from the so-called hypervirulent strains that can cause highly invasive infections such as liver abscess but are usually drug sensitive [2, 3]. The antimicrobial resistance (AMR) and/or virulence determinants possessed by the associated bacteria are generally found on mobile genetic elements (MGEs) that transmit between *K*. *pneumoniae* cells via horizontal gene transfer (HGT) [4]. These MGEs, most typically plasmids and integrative and conjugative elements (ICEs), are therefore important constituents of the accessory genome that imbue *K*. *pneumoniae* organisms with their distinct HA or CA clinical profiles.

It is apparent that a wide diversity of *K*. *pneumoniae* can cause infections in hospitalised patients [3, 5, 6] and that basic pathogenicity factors such as lipopolysaccharide, capsular polysaccharide, type 3 fimbriae and the siderophore enterobactin (Ent) are common to all *K*. *pneumoniae* and conserved in the chromosome as core genes [1, 3]. However, enhanced virulence or ‘hypervirulence’ is associated with specific capsular serotypes (K1, K2, K5) and with MGE-encoded accessory genes that are much rarer in the *K*. *pneumoniae* population [3]. Of particular importance are those encoding additional siderophore systems, namely yersiniabactin (Ybt) [3, 7, 8], aerobactin (Iuc) [9] and salmochelin (Iro) [10].

Synthesis of acquired siderophores contributes to *K*. *pneumoniae* virulence via multiple mechanisms. However, iron assimilation via the conserved siderophore Ent is hampered by human neutrophils and epithelial cells through the secretion of lipocalin-2 (Lcn2), which binds, and thus inhibits bacterial uptake of, iron-loaded Ent [11]. Ybt, Iro and Iuc on the other hand are not subject to Lcn2 binding; Iro is a glycosylated derivative of Ent, while Ybt and Iuc possess an entirely distinct structure from Ent. The ability of salmochelin to counter Lcn2 binding is important for bacterial growth and has been shown to correlate with enhanced virulence in a mouse sepsis model [12]. The association between aerobactin and virulence has long been recognised, with multiple studies demonstrating its key role in an increased iron acquisition, bacterial growth and/or virulence in various murine models, human ascites fluid and blood [9, 13–15]. Even in strains that possess all four siderophore-encoding loci, Iuc appears to play the most critical role in virulence both in vitro and in vivo [13] and serves as an important biomarker for identifying hypervirulent isolates [16].

**Fig. 1:**
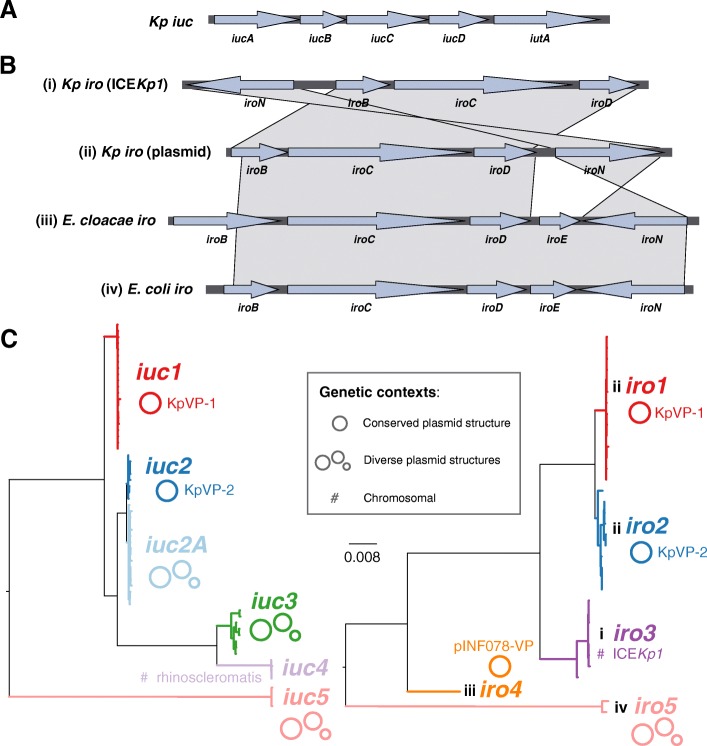
Aerobactin and salmochelin locus variants found in *Klebsiella pnuemoniae*. **a** A single aerobactin (*iuc*) locus structure was found in *K*. *pneumoniae*. **b** Four different structures of the salmochelin (*iro*) locus were found in *K*. *pneumoniae* (i–iv). Note two of these are typical of structures found in other species (iii in *Enterobacter cloacae*, iv in *Escherichia coli*). **c** Maximum likelihood phylogenetic trees inferred from *iuc* and *iro* sequence types (AbSTs and SmSTs) identified in *K*. *pneumoniae* genomes. Phylogenetic lineages discussed in the text are labelled and their mobility indicated; nucleotide divergence within and between lineages is given in Additional files 8 and 9. *Iro* locus structures associated with each lineage are labelled i–iv, as defined in panel **b**

In *K*. *pneumoniae*, Ybt biosynthesis is encoded by the *ybt* locus, which is typically located on a chromosomal ICE known as ICE*Kp* (of which there are at least 14 distinct variants) and was recently also reported on plasmids [7, 8, 17]. A screen of 2500 *K*. *pneumoniae* genomes showed *ybt* to be prevalent in one third of the sequenced population and associated with hundreds of putative ICE*Kp* acquisition events across the chromosomes of both hypervirulent and MDR lineages [8]. In contrast, Iuc and Iro synthesis is encoded by loci (*iuc* and *iro*, depicted in Fig. 1), that are typically co-located on the so-called ‘virulence plasmids’ of *K*. *pneumoniae*. The best characterised virulence plasmids are the 224 kbp plasmid pK2044 from serotype K1, sequence type (ST) 23 strain NTUH-K2044 [18]; the 219 kbp plasmid pLVPK from K2, ST86 strain CG43 [19]; and the 121 kbp plasmid Kp52.145pII from serotype K2, ST66 strain Kp52.145 (strain also known as 52145 or B5055; plasmid also known as pKP100) [9, 20]. These plasmids also carry additional virulence determinants including *rmpA* genes that upregulate capsule production, conferring a hypermucoid phenotype that is considered a hallmark of hypervirulent strains [21], other gene clusters associated with iron uptake and utilisation and other loci encoding resistance to heavy metals such as copper (*pco-pbr*), silver (*sil*) and tellurite (*ter*) [4]. In addition to the virulence plasmid-encoded *iro* and *rmpA* genes, the ST23 strain NTUH-K2044 also carries a chromosomal copy of *iro* and *rmpA* located within ICE*Kp1* [7]; however, this is not a typical feature of ST23 [22]*.*

The majority of *K*. *pneumoniae* lineages associated with liver abscess and other invasive community-acquired infections (e.g. clonal group (CG) 23, CG86, CG380) carry virulence plasmids encoding *iro*, *iuc* and *rmpA* [3, 9, 16, 23–25]. However, while virulence and AMR genes are both transmitted within the *K*. *pneumoniae* population via plasmids, until recently, these plasmids have mainly been segregated in non-overlapping populations such that the virulence plasmids encoding *iuc* and *iro* have rarely been detected in MDR populations that cause HA infections and outbreaks [3, 4, 26]. However, the virulence plasmid Kp52.145pII has been shown experimentally to be mobilisable [21], and there are emerging reports of MDR clones such as ST11, ST147 and ST15 acquiring virulence plasmids [27, 28]. The combination of hypervirulence and MDR can result in invasive infections that are very difficult to treat. This can result in dangerous hospital outbreaks; for example, an aerobactin-producing carbapenemase-producing ST11 strain recently caused a fatal outbreak of ventilator-associated pneumonia in a Chinese intensive care unit, with 100% mortality [27, 29]. AMR plasmids are also occasionally acquired by ST23 and other hypervirulent *K*. *pneumoniae* clones [25, 30, 31].

The ease with which virulence plasmids spread in the *K*. *pneumoniae* population poses a significant global health threat, highlighting the importance of understanding and monitoring the movement of these loci between different strains and clones. Here, we investigate the diversity of aerobactin and salmochelin synthesis loci in 2733 *K*. *pneumoniae* complex genomes, aiming to understand the diversity and distribution of these virulence loci in the population and to develop a framework for their inclusion in genomic surveillance efforts.

## Methods

### Bacterial genome sequences

2733 genomes of the *K*. *pneumoniae* complex, including isolates collected from diverse sources and geographical locations, were analysed in this study (see Additional file 1). The genomes represent a convenience sample of our own isolate collections from clinical and species diversity studies [5, 8, 22, 32], as well as sequences that were publicly available in GenBank or via the NCTC 3000 project (https://www.sanger.ac.uk/resources/downloads/bacteria/nctc/) at the commencement of the study (June 2017). The majority of these genomes were also included in our previous genome study screening for yersiniabactin and colibactin [8].

For *n* = 1847 genomes (see Additional file 1), Illumina short reads were available, and these were used to generate consistently optimised de novo assembly graphs using Unicycler v0.3.0b with SPAdes v3.8.1 [33, 34]. The remaining *n* = 886 genomes were publicly available only in the form of draft genome assemblies, i.e. with no reads available for direct analysis. All genome assemblies were re-annotated using Prokka [35] to allow for standardised comparison. All genomes were assigned to species by comparison to a curated set of Enterobacteriaceae genomes using mash (implemented in Kleborate, https://github.com/katholt/Kleborate); this confirmed 2503 *K*. *pneumoniae*, 12 *Klebsiella quasipneumoniae* subsp. *quasipneumoniae*, 59 *K*. *quasipneumoniae* subsp. *similipneumoniae*, 158 *Klebsiella variicola* and 1 *Klebsiella quasivariicola* (Additional file 1).

### Long-read sequencing of isolates

Three isolates in our own collection (INF078, INF151, INF237) carried novel *iuc* and/or *iro* plasmids identified from short-read Illumina data. We subjected these to long-read sequencing using a MinION R9.4 flow cell (Oxford Nanopore Technologies (ONT)) device in order to resolve the complete sequences for the relevant plasmids. Overnight cultures of each isolate were prepared in LB broth at 37 °C, and DNA extracted using Agencourt Genfind v2 (Beckman Coulter) according to a previously described protocol (doi: 10.17504/protocols.io.p5mdq46). Sequencing libraries were prepared using a 1D ligation library (SQK-LSK108) and native barcoding (EXP-NBD103) as previously described [22, 36]. The resulting reads were combined with their respective Illumina reads to generate a hybrid assembly using our Unicycler software v0.4.4-beta [33, 36]. Note this approach uses ONT reads to bridge together contig sequences constructed from Illumina data, followed by consensus base call polishing with both types of reads. Annotations for the hybrid assemblies were generated as described above, and the annotated sequences submitted to GenBank under accession numbers QWFT01000001-QWFT01000009, and CP032831-CP032838 (Additional files 1, 2 and 3).

### Multi-locus sequence typing

Chromosomal sequence types were determined for each genome assembly using the BIGSdb-*Kp* seven-locus multi-locus sequence typing (MLST) scheme [37] screened using Kleborate (https://github.com/katholt/Kleborate). A novel ST (ST3370) was identified and added to the BIGSdb-*Kp* MLST database.

To facilitate the development of MLST schemes for the aerobactin and salmochelin biosynthesis loci *iuc* and *iro*, alleles for genes belonging to each locus (i.e. *iucABCD*, *iutA*; and *iroBCDN*; respectively) from genomes with ‘typeable’ loci (defined as those in which all genes in the locus had high-quality consensus base calls when mapping with SRST2) were extracted by comparison to known alleles in the BIGSdb-*Kp* virulence database (http://bigsdb.pasteur.fr/klebsiella/klebsiella.html) [25], using SRST2 v0.2.0 [38] to screen Illumina read sets where available and BLAST+ v2.2.30 to screen assemblies. Incomplete, ‘non-typeable’ *iro* and *iuc* loci were excluded from the MLST scheme (marked NT in Additional file 1). Each unique combination of alleles was assigned an aerobactin sequence type (AbST) or salmochelin sequence type (SmST), defined in Additional files 4 and 5. The AbST and SmST schemes, profiles and corresponding alleles are also available in the BIGSdb-*Kp* database and in the Kleborate Github repository (see links above).

### Identification of other genes of interest and genetic context of *iuc* and *iro* loci

Capsule (K) loci were identified in each assembled genome using Kaptive [39]. *RmpA* gene copy number was determined by BLASTn search of all genome assemblies using the *rmpA* and *rmpA2* sequences from pK2044 (GenBank accession AP006726.1) as queries with > 90% coverage and > 90% nucleotide identity. Similarly, BLASTn was used to screen the genome assemblies for the IncFIB_K_ *repA* sequence from virulence plasmids pK2044 and Kp52.145 pII (GenBank accession FO834905.1), with IncFIB_K_ presence defined as > 90% coverage and > 80% nucleotide identity to these query sequences (to ensure inclusion of known IncFIB_K_ sequences while excluding detection of non-FIB_K_ sequences such as the IncFIB sequences frequently detected in other Enterobacteriaceae bacteria**)**. IncFII replicons were identified using BLASTn search of the PlasmidFinder database [40].

Assemblies of all *iuc*+ or *iro*+ genomes were manually inspected to determine whether the loci of interest were located on the chromosome or on previously described virulence plasmids (pK2044 and Kp52.145pII). This confirmed most to be located in the chromosome (*iro3* in ICE*Kp1* or *iuc4* in the subspecies *rhinoscleromatis* lineage) or one of the known plasmids. For the remaining genomes, annotated contigs containing the *iuc* and/or *iro* loci were checked for known chromosomal or plasmid features, aided by BLASTn searching against the NCBI non-redundant nucleotide database and inspection of the assembly graphs using Bandage v0.8.0 [41].

### Phylogenetic analyses

Maximum likelihood phylogenetic trees capturing the relationships between AbSTs or SmSTs were constructed by aligning the allele nucleotide sequences corresponding to each sequence type within each scheme using MUSCLE v3.8.31 [42] then using each of the two alignments (one for AbSTs, one for SmSTs) as input for phylogenetic inference in RAxML v7.7.2 [43]. For each alignment, RAxML was run five times with the generalised time-reversible model and a gamma distribution, and the trees with the highest likelihood were selected. Lineages were defined as monophyletic groups of AbSTs or SmSTs, which were each associated with a unique MGE structure; STs within lineages shared ≥ 2 alleles (for SmSTs) or ≥ 3 alleles (for AbST), whereas no alleles were shared between lineages.

Maximum likelihood phylogenies were similarly constructed for (i) aerobactin and salmochelin locus alignments populated by sequences extracted from BLAST hits amongst representatives of the wider Enterobacterales order (representatives listed in Additional file 6) and (ii) IncFIB_K_ replicon sequence alignments constructed by mapping *iuc*-positive (*iuc*+) and *iro*-positive (*iro*+) genomes to a reference IncFIB_K_ sequence (coordinates 128130 to 132007, spanning *repA* to *sopB*, of the pK2044 plasmid sequence; GenBank accession AP006726.1).

### Plasmid comparisons

Twelve representative plasmids (10 complete, including *n* = 3 generated from hybrid long- and short-read assemblies detailed above, and 2 partial) were chosen for comparative analysis (these are available as a set in FigShare under doi: 10.6084/m9.figshare.6839981; and see Additional file 2 for list of sources and GenBank accession numbers). Six of these representative plasmids were sourced from the NCTC 3000 project (https://www.sanger.ac.uk/resources/downloads/bacteria/nctc/). As no complete plasmid sequences from *K*. *pneumoniae* were available with *iuc5*, we used plasmid p3PCN033 from *E*. *coli* as the reference for *iuc5*. We consider this appropriate in the circumstances since the *K*. *pneumoniae iuc5* plasmids shared with p3PCN033 the IncFII replicon (native to *E*. *coli*) and the *iuc* and *iro* sequences and structural variants typical of *E*. *coli*; the *iuc5* contigs from *K*. *pneumoniae* showed 99.19–99.95% sequence identity with p3PCN033, and IncFII plasmids while considered native to *E*. *coli* have been detected in *Klebsiella pneumoniae* alongside other Enterobacteriaceae members [44, 45].

The representative plasmid sequences were compared using Mauve v2.4.0 [46], in order to identify homology blocks conserved amongst subsets of the plasmids. BLASTn comparisons of related plasmids were plotted using GenoPlotR v0.8.7 package [47] for R. All *iuc*+ or *iro*+ genomes were mapped against all 12 representative plasmids in order to calculate the coverage of each plasmid in each genome. This was done using Bowtie2 v2.2.9 [48] to map Illumina reads where available, and 100 bp reads simulated from draft assemblies where raw sequence reads were not available, using the RedDog pipeline (https://github.com/katholt/RedDog). For every gene annotated within each reference plasmid, the proportion of isolates within each group of genomes sharing the same *iuc*/*iro* lineage carrying the gene was calculated using the gene presence/absence table reported by RedDog (presence defined as ≥ 95% of the length of the gene being covered by at least five reads) and plotted as circular heatmaps using ggplot2 in R (using geom_tile to achieve a heatmap grid and polar_coord to circularise).

## Results

### Prevalence of *iuc* and *iro* in *K*. *pneumoniae*

*Iuc* and *iro* were detected only in *K*. *pneumoniae* genomes, and not in other members of the *K*. *pneumoniae* species complex. Of the 2503 *K*. *pneumoniae* genomes screened, *iuc* was detected in 8.7% (*n* = 217) and *iro* in 7.2% (*n* = 181; listed in Additional file 1, excluding those with a partial *iro* locus as discussed below). The presence of intact *iro* and *iuc* loci was strongly associated (odds ratio (OR) 711, 95% confidence interval (CI) 386–1458, *p* < 1 × 10^−16^), co-occurring in 162 genomes (6.5% of the genomes tested). The *iro* locus appears to be susceptible to deletion; partial *iro* loci were observed in *n* = 50 *K*. *pneumoniae* isolates (noted as iro* in Additional file 1), mostly those that were isolated from historical collections prior to 1960. Of 39 isolates collected prior to 1960 and with any *iro* genes present, 36 (92%) carried deletion variants of the locus, compared to 4/163 (2.5%) amongst isolates from 1975 onwards (OR 416, 95% CI 88–3297, *p* < 2 × 10^−16^). As expected, the presence of *iuc* and *iro* was each strongly associated with the presence of *rmpA*, with 157 genomes carrying all three loci (excluding partial *iro*). A total of 238 genomes (9.5%) carried *rmpA* genes: *n* = 110 (4.4%) carried one, *n* = 127 (5.1%) carried two, and a single genome, ST23 NTUH-K2044, carried three (as described previously [7, 18], see Additional file 1).

### Genetic diversity of *iuc* and *iro* in *K*. *pneumoniae*

Next, we explored nucleotide diversity of the genes comprising the *iro* and *iuc* loci in *K*. *pneumoniae.* The five genes comprising the *iuc* locus (Fig. 1a) and four genes of the *K*. *pneumoniae* forms of the *iro* locus (Fig. 1b) were screened for sequence variation, and each unique gene sequence variant was assigned an allele number. Of the *n* = 209 genomes carrying a typeable *iuc* locus, 62 unique *iuc* allele combinations were observed and assigned a unique aerobactin sequence type or AbST (see Additional file 4 for AbST definitions and Additional file 1 for AbSTs assigned to each genome). The *iutA* alleles present in the *iuc* locus showed > 28% nucleotide divergence from a core chromosomal paralog of *iutA* encoding a TonB-dependent siderophore receptor (positions 2043670–2045871 in NTUH-K2044), which we observed in 96.4% of all genomes; the alleles of this core chromosomal gene are not included in the aerobactin MLST scheme. Typeable *iro* loci were identified in *n* = 164 genomes, comprising 35 unique salmochelin sequence types or SmSTs (defined in Additional file 5, see Additional file 1 for SmSTs assigned to each genome). Maximum likelihood phylogenetic analyses of the AbST and SmST sequences, and their translated amino acid sequences, revealed five highly distinct *iuc* lineages and five *iro* lineages (labelled *iro1*, *iro2* etc.; see Fig. 1c, Additional file 7). Nucleotide divergence between lineages was 1–11% (20–1000 substitutions), and no alleles were shared between lineages (Additional files 8 and 9). Nucleotide divergence within lineages was low, with mean divergence of 0.001–0.40% (*iro*) and 0.013–0.50% (*iuc*) (Additional files 8 and 9) and at least two (*iro*) or three (*iuc*) shared alleles between members of the same lineage. Of note, the *iro4*, *iro5* and *iuc5* loci were quite distant from other lineages (each showing > 5.5% nucleotide divergence from all other lineages vs < 4.6% divergence amongst the other lineages; Fig. 1, Additional files 8 and 9). Comparison to *iuc* and *iro* genes present in other bacteria (all of which were members of the order Enterobacterales, see Additional files 10 and 11), and the presence of the additional *iroE* gene that we observed in other bacteria (all of which were members of family Enterobacteriaceae, see Fig. 1b), suggests that these more distant lineages derive from outside *Klebsiella*, most likely *Enterobacter* (*iro4*) and *E*. *coli* (*iro5*, *iuc5*). Note that genotyping of *rmpA* was not performed since most *rmpA*-positive genomes carry two copies of the gene, which complicates allele typing from short-read data; however, *rmpA* copy number per genome is reported in Additional file 1.

### Mobile genetic elements associated with *iuc* and *iro* loci

Inspection of the genetic context surrounding the *iuc* and *iro* sequences revealed that the various *iuc* and *iro* lineages were associated with distinct MGEs, with the exception of *iuc4* which was restricted to the chromosome of *K*. *pneumoniae* subspecies *rhinoscleromatis* (ST67) (Fig. 1c, Table 1). Most common were *iuc1* and *iro1*; these were both associated with pK2044-like plasmids (hereafter called KpVP1-1, see below) and the presence of two *rmpA* genes and accounted for 74% of all *iuc*+*iro*+ genomes. These were followed by *iuc2* and *iro2*, which were associated with Kp52.145 pII-like plasmids (hereafter called KpVP-2, see below), the presence of one *rmpA* gene, and accounted for 14% of all *iuc*+*iro*+ genomes. A sister clade of *iuc2*, which we named *iuc2a*, was associated with diverse plasmids that shared some homology with Kp52.145 pII (36–70% coverage, 99% nucleotide identity). Most *iuc2a*+ isolates carried a single *rmpA* gene (*n* = 38, 88.4%), and all lacked an intact *iro* locus (*n* = 26, 60.5% had a partial *iro* locus). Lineage *iuc3* was related to the *iuc4* lineage encoded on the *rhinoscleromatis* chromosome but was present on novel plasmids. *Iro3* was located within the chromosomally integrated ICE*Kp1*, along with *rmpA*. Four genomes carried *iuc5* (two of these also carried *iro5*; all lacked *rmpA*). The *iuc5* sequences were distantly related to *iuc1* and *iuc2* (> 8.9% nucleotide divergence) but were identical to sequences found in *E*. *coli* and located on contigs that matched closely to *E*. *coli* AMR plasmids (e.g. strain PCN033 plasmid p3PCN033, accession CP006635.1 [49], which showed > 99% nucleotide identity to the best assembled of *iuc5*+ *K*. *pneumoniae* contigs). *Iro4* was identified in a single genome (which lacked *rmpA*) and was > 6.1% divergent from *iro1* and *iro2* sequences. Its closest known relatives are *iro* sequences present in the chromosomes of *Enterobacter cloacae* and *Enterobacter hormaechei* (strains AR_0065, accession CP020053.1, and 34977, accession CP010376.2, respectively; 95% identity). Lineages *iro4* and *iro5* follow the gene configuration typical of non-*K*. *pneumoniae* Enterobacteriaceae *iro* loci, from which the *K*. *pneumoniae iro1*, *iro2* and *iro3* differ by lack of *iroE* and inversion of *iroN* (see Fig. 1b).

**Table 1:** Summary of *iuc* and/or *iro* plasmid lineages

Lineage(s)	*N*	Mobile genetic element	Reference(s)
*iuc1* (+ *iro1*)	121 (119)	*K*. *pneumoniae* VP-1, type I IncFIB_K_ + IncHI1B, *rmpA*+*rmpA2*	pK2044 (accession AP006726.1)
*iuc2* (+ *iro2*)	23 (23)	*K. pneumoniae* VP-2, type II IncFIB_K_, *rmpA*	Kp52.145 plasmid II (accession FO384905.1)
*iuc2a*	43	Novel, diverse plasmids IncFIB_K_ + other IncF replicons, sometimes IncFII *tra*	Many distinct types Novel examples: pINF151_01-VP (accession QWFT01000004), pINF237_01-VP (accession CP032834)
*iuc3*	11	Novel, diverse plasmids IncFIB_K_ + IncFII *tra*	NCTC11676, NCTC11697
*iuc4*	7	Chromosomal integration	*K. pneumoniae rhinoscleromatis*, e.g. strain SB3432 (accession FO203501.1)
*iuc5* (+*iro5*)	4 (2)	*E*. *coli* IncFII *tra* plasmid *E. coli iroBCDEN* + AMR	*E. coli* strain PCN033 plasmid p3PCN033 (accession CP006635.1)
*iro3*	16	Chromosomal ICE*Kp1*	*K. pneumoniae* NTUH-K2044 ICE*Kp1* (accession AB298504.1)
*iro4*	1	Novel plasmid IncFIB_K_ + IncFII *tra* *E*. *cloacae*/*E. hormaechei iroBCDEN* (× 13 copies)	pINF078-VP (accession CP032832)

**Fig. 2:**
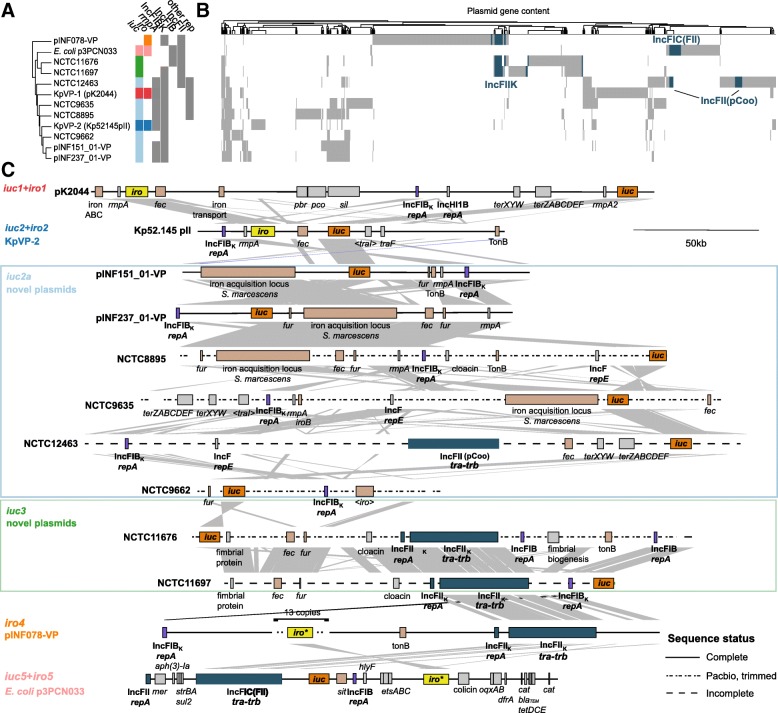
Plasmid variants associated with different *iro* and/or *iuc* lineages identified amongst *K*. *pneumoniae.* **a** Clustering of the 12 reference plasmids based on gene content, annotated with the presence of *iuc* and *iro* lineages (coloured as in panel **b** and Fig. 1c), *rmpA,* IncFIB_K_, IncFIB, IncFII and/or other plasmid replicon types. **b** Gene content matrix for reference plasmids; columns correspond to protein-coding sequences that are > 10% divergent from one another. IncFII *tra-trb* conjugal transfer region genes are coloured blue, to highlight the divergent forms of this region and labelled with the closest IncFII type as detected by PlasmidFinder. **c** Genetic maps for the reference plasmids. The positions of key loci involved in core plasmid functions (bold), virulence (*iro* highlighted in yellow, *iuc* in dark orange and other loci involved in iron acquisition/transport in light orange) and antimicrobial resistance are indicated. Grey shading indicates homology blocks sharing > 60% nucleotide identity

To examine the gene content and replicon differences between the various *K*. *pneumoniae* plasmids associated with *iuc* and/or *iro*, 12 representative plasmids associated with the various lineages were selected for comparison (Fig. 2, Additional file 2). These include six complete *K*. *pneumoniae* plasmid sequences identified from finished genomes: *iuc1*/*iro1* (*n* = 1), *iuc2*/*iro2* (*n* = 1), *iuc2a* (*n* = 3), *iuc3* (*n* = 1); three novel complete *K*. *pneumoniae* plasmid sequences that we generated for this study, carrying *iuc2a* (*n* = 2) and *iro4* (*n* = 1); and two large contigs that we identified from public *K*. *pneumoniae* genome data representing partial sequences for additional plasmids carrying *iuc2a* (*n* = 1) and *iuc3* (*n* = 1) (Fig. 2). The *K*. *pneumoniae* genomes in which *iuc5*/*iro5* were identified were available only as draft assemblies deposited in public databases, and the associated plasmid sequences were fragmented in these assemblies; hence, we used *E*. *coli* strain PCN033 plasmid p3PCN033 [49] as the representative for *iuc5*/*iro5*. The representative plasmid sequences differed substantially in their structure and gene content between and within the different lineages (Fig. 2b, c).

**Fig. 3:**
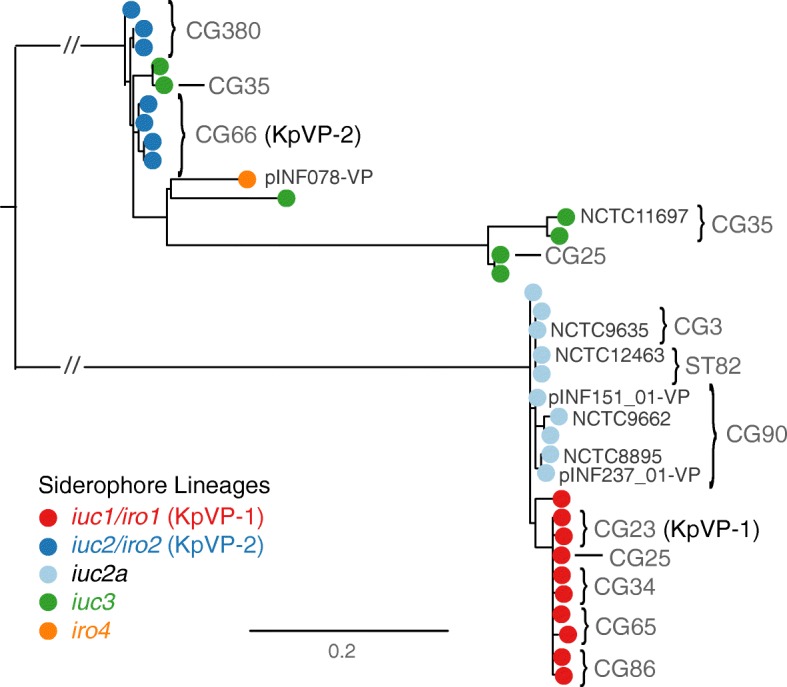
Maximum likelihood phylogeny of representative IncFIB_K_ replicon sequences from isolates with *iuc*/*iro* plasmids. Each tip represents a unique IncFIB_K_ replicon sequence (spanning *repA*, *oriT*, *sopAB*), coloured according to the *iro*/*iuc* lineage carried by the corresponding isolates as per inset legend. IncFIB_K_ sequences found in the representative plasmid sequences (shown in Fig. 2 and listed in Additional file 2) are labelled; tips/subclades are also annotated to indicate those found in common clonal groups (CG; see Fig. 5)

All representative *iuc* or *iro* plasmids harboured an IncFIB_K_ (*n* = 9) or IncFIB (*n* = 3) replicon, including the *repA* replication gene, *oriT* origin of transfer and *sopAB* partitioning genes (presence of these replicons in each plasmid is indicated purple in Fig. 2c and listed in Additional file 2). The IncFIB_K_ replicon was present in *n* = 202/208 (97%) of isolates with plasmid-encoded *iuc* or *iro*, including 100% of *iuc1*/*iro1*, *iuc2*/*iro2*, *iuc2a* and *iro4* isolates, and 82% of *iuc3* isolates. Each of these *iuc*/*iro* lineages was associated with a unique sequence variant of the IncFIB_K_ replicon (see tree in Fig. 3 and nucleotide identity with the IncFIB_K_ *rep* sequences from KpVP-1 and KpVP-2 listed in Additional file 1), supporting the segregation of the *iuc* and *iro* loci with distinct FIB_K_ plasmid backbones. However, the IncFIB_K_ replicon was also widely detected amongst isolates that do not carry *iro* and *iuc* (77% of all *K*. *pneumoniae* genomes and 69% amongst other species in the complex; see Additional file 1), including MDR *K*. *pneumoniae* lineages such as CG258, and is known to be associated with AMR plasmids [44, 50]. IncFIB replicons, which are common amongst *E*. *coli* and display > 39% nucleotide divergence from the IncFIB_K_ replicon, were found in all *K*. *pneumoniae* isolates carrying the *E*. *coli* variant *iuc5* (100%) and also detected in two isolates carrying *iuc3* plasmids (18%; marked in Fig. 2a, c), suggesting the transfer of these *iuc* variants into *K*. *pneumoniae* via such plasmids.

**Fig. 4:**
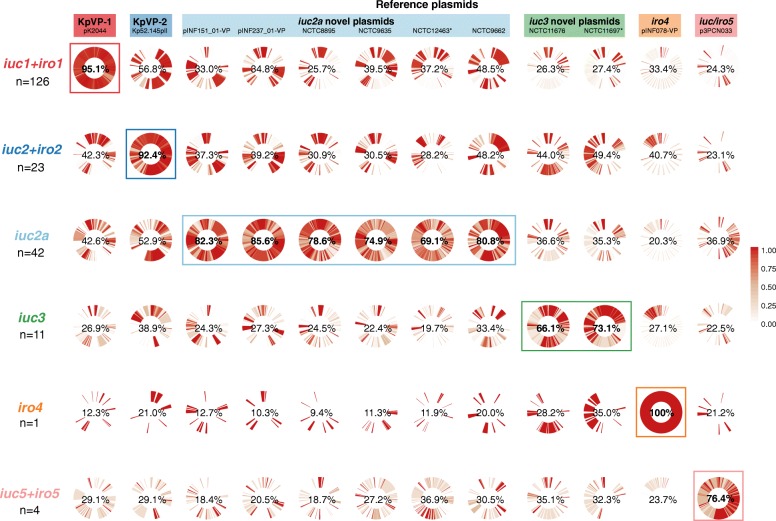
Conservation of reference plasmid genes amongst isolates with plasmid-associated *iuc*/*iro* lineages. Cells show circularised heatmaps indicating the frequency of each gene in a given reference plasmid (column), amongst isolates that contain a given *iro* and/or *iuc* lineage (row). Around each circle, genes are ordered by their order in the corresponding reference plasmid. Percentages in the middle of each cell indicate the mean coverage of the reference plasmid sequence (column), amongst isolates belonging to each *iro*/*iuc* lineage (row); bold labels and boxes highlight groups of isolates carrying the same *iuc*/*iro* lineage as the reference plasmid. ‘*’ indicates the two plasmids represented by incomplete plasmid sequences

**Fig. 5:**
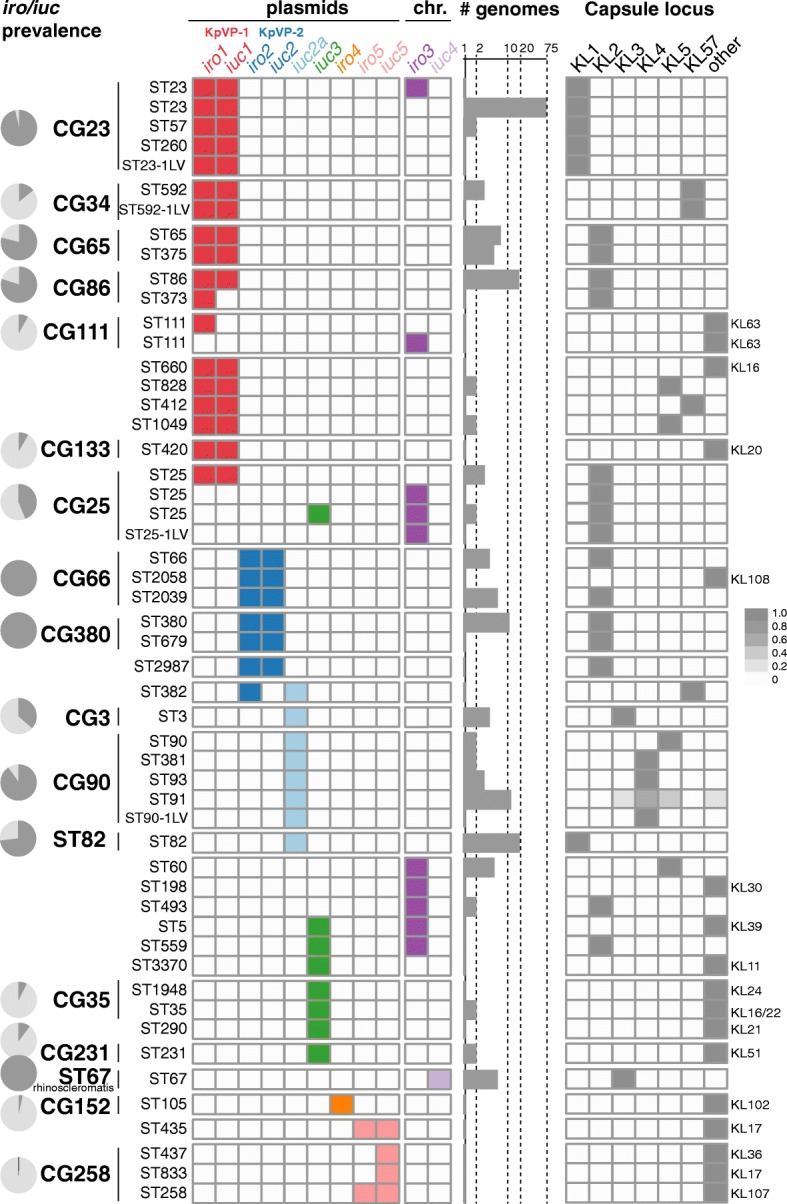
Distribution of plasmid and chromosomal variants of *iro* and *iuc* and capsule locus (KL) types amongst *K*. *pneumoniae* clones. Rows indicate sequence types (STs, as labelled) that contain ≥ 1 genome in which *iro* and/or *iuc* was detected; vertical lines indicate STs belonging to the same clonal group (CG) as labelled. Pie charts indicate prevalence of *iro* and/or *iuc* within common *K*. *pneumoniae* lineages. The detection of individual *iro* and *iuc* lineages within each *K*. *pneumoniae* ST is indicated in the grid, coloured as per Fig. 1. Bar plots indicate sample size (number of genomes per ST; note log_10_ scale). Heatmap on the right indicates prevalence of capsule (K) locus types in each *K*. *pneumoniae* ST, coloured as per inset legend. Individual columns are included for K types that are common amongst virulent clones; where other K types were detected, these are represented in the ‘other’ column, and the relevant K type for that ST is labelled to the right

In order to explore structural conservation of plasmids amongst isolates with each *iro* or *iuc* lineage, we mapped the sequence data from all isolates carrying either of these loci against the 12 representative plasmid sequences (Fig. 4). This revealed that plasmid structures were largely conserved amongst isolates sharing the same *iuc* or *iro* lineages, although plasmids associated with *iuc2a* and *iuc3* showed more diversity than others (Fig. 4 and see below). The distribution of *iuc* and *iro* variants with respect to the clonal group of the host strain, identified by chromosomal MLST, shows that each follows quite distinct patterns of dissemination in the *K*. *pneumoniae* population (Fig. 5).

### *Iuc*/*iro* lineages 1 and 2 are associated with two dominant *K*. *pneumoniae* virulence plasmids, KpVP-1 and KpVP-2

*Iuc*/*iro* lineages 1 and 2 accounted for 64% of *K*. *pneumoniae* isolates carrying any aerobactin or salmochelin synthesis loci, and 88% of isolates carrying both. While it was not possible to resolve the complete sequences for all plasmids associated with these lineages, read mapping to pK2044 and Kp52.145 pII reference sequences strongly supported the presence of pK2044-like plasmids in *iro1*+*iuc1*+ genomes (mean plasmid coverage of 95.1%, range 28.8–100%; see Fig. 4) and Kp52.145 pII-like plasmids in *iro2*+*iuc2*+ genomes (mean plasmid coverage of 92.4%, range 87.2–100%; see Fig. 4). There were limited homologous regions shared between the two plasmids (Fig. 2), including the *iro*, *iuc*, *rmpA* and *fec* loci, and the IncFIB_K_ replicon (Additional file 12). These shared regions were largely conserved across all isolates carrying *iuc*/*iro* lineages 1 or 2; the remaining regions unique to either pK2044 or Kp52.145 pII were largely conserved amongst the isolates that carried lineage 1 or 2 loci, respectively (Fig. 4). Notably, the loci encoding heavy metal resistances against copper (*pbr-pco*), silver (*sil*) and tellurite (*terXYW* and *terZABCDEF*) were highly conserved amongst lineage 1 isolates but not present in any of the lineage 2 isolates (Additional file 12). As noted above, *iuc*/*iro* lineages 1 and 2 were also each associated with a distinct variant of the IncFIB_K_ replicon sequence (Fig. 3). Hence, we define pK2044-like plasmids carrying *iuc1* and *iro1* loci as *K*. *pneumoniae* virulence plasmid type 1 (KpVP-1), with reference plasmid pK2044, and Kp52.145 pII-like plasmids carrying *iuc2* and *iro2* loci as *K*. *pneumoniae* virulence plasmid type 2 (KpVP-2). Both plasmid types typically carry at least one copy of *rmpA*; neither one carries genes associated with conjugation; hence, we assume they are not self-transmissible.

KpVP-1 and KpVP-2 showed distinct distributions within the *K*. *pneumoniae* population. KpVP-1 was present in 5.0% of all isolates and accounted for 74% of *iuc*+*iro*+ isolates. The KpVP-1 reference plasmid pK2044 originated from an ST23 isolate (CG23), and KpVP-1 was strongly associated with this and two other well-known hypervirulent clones CG65 and CG86, in which it was present at high prevalence (ranging from 79.0 to 96.4%, see Fig. 5). KpVP-1 was also detected at low frequencies in other clones, including CG34, CG111, CG113 and CG25, suggesting it is mobile within the *K*. *pneumoniae* population (Fig. 5). KpVP-2 was present in 0.96% of all isolates and accounted for 14% of *iuc*+*iro*+ isolates. The KpVP-2 reference plasmid Kp52.145 pII originated from an ST66 isolate, and KpVP-2 was present in all isolates of the associated clonal group CG66 (*n* = 11) and also all isolates of CG380 (*n* = 12) (Fig. 5).

### An *iuc* lineage 2 variant (*iuc2a*) is associated with diverse plasmids with a KpVP-1-like IncFIB_K_ replicon

*Iuc2a* was identified in 43 isolates largely belonging to three clonal groups (ST3, *n* = 4; CG90, *n* = 19; ST82, *n* = 19; ST382, *n* = 1; see Fig. 5), with the majority (*n* = 38, 88.4%) from the historical NCTC or Murray collections and isolated between 1932 and 1960 (Additional file 1). One of these isolates also carried *iro2* in addition to *iuc2a*, which in all other instances was only observed with *iuc2* on KpVP-2*.* Provenance information was available for only 12 of the *iuc2a*+ isolates (1 ST3, 9 CG90, 2 ST82); all of which originated from the human respiratory tract (3 nose, 1 throat, 7 sputum and 2 NCTC isolates recorded only as a respiratory tract). We used long-read sequencing to resolve plasmids in two novel *iuc2a*+ isolates from our own collection, INF151and INF237, which were both CG90 Australian hospital sputum isolates (summarised in Additional file 3). This yielded IncFIB_K_ plasmids in each genome, of size 138.1 kbp and 133.7 kbp, respectively (accessions: pINF151_01-VP, QWFT01000004; pINF237_01-VP, CP032834). Both plasmids carried *iuc2a* and one *rmpA* gene, but they differed slightly from one another in structure and gene content and differed substantially from the three complete *iuc2a*+ plasmid sequences available from NCTC isolates (ST3 and CG90; see Figs. 2 and 4). Only one of these plasmids (from NCTC 12463; incomplete) carried a conjugative transfer region (IncFII); hence, we predict most are not self-transmissible. Mapping of *iuc2a*+ genomes to each of the five representative *iuc2a*+ plasmid sequences indicated a degree of conservation between plasmids in isolates belonging to the same *K*. *pneumoniae* clone, but none particularly well conserved across all *iuc2a*+ isolates (Fig. 4, Additional file 13). However, all *iuc2a*+ isolates formed a tight monophyletic cluster in the IncFIB_K_ replicon tree (Fig. 3), consistent with recent shared plasmid ancestry followed by frequent structural and gene content changes. Notably, the *iuc2a*-associated IncFIB_K_ replicon sequences were closely related to those of KpVP-1 and distant from those of KpVP-2; hence, we hypothesise that *iuc2a* plasmids share an ancestor that was a mosaic including *iuc2*-related sequences from KpVP-2 and IncFIB_K_ replicon sequences from KpVP-1.

### *Iuc* lineage 3 is mobilised by diverse plasmids carrying the IncFII_K_ conjugative transfer region

Lineage *iuc3* was detected in 11 isolates from diverse sources and chromosomal STs (Fig. 5) and was associated with three related variants of the IncFIB_K_ replicon (Fig. 3). We identified one complete and one near-complete *iuc3* plasmid sequences: a complete 189.8 kb plasmid from NCTC 11676 (isolated 1979, ST290) and a 155.4 kb contig from NCTC 11697 (isolated 1984, ST3370) (Fig. 2). The plasmids share around half of their gene content (96 kbp), including the IncFII_K_ *tra-trb* conjugative transfer machinery, a fimbrial protein and the *fec* iron acquisition system in addition to *iuc3* (Figs. 2 and 4, Additional file 2). Mapping to these sequences showed all *iuc3*+ isolates carried related plasmids with an IncFII_K_ *tra-trb* transfer region (Fig. 4, Additional file 12).

### Complete sequence of an *iro4* plasmid

Lineage *iro4* was identified in a single hospital UTI isolate INF078 (ST105) from Australia, whose genome sequence we completed using long reads (replicons summarised in Additional file 3). Hybrid assembly using short and long reads resolved a 399,913 kbp plasmid, pINF078-VP (accession CP032832) which carried multiple copies of *iro4*, the IncFIB_K_ replicon (similar to the KpVP-2 variant, see Fig. 5) and the IncFII_K_ replicon and *tra-trb* transfer region (Fig. 2). As noted above, the *iro4* locus is more closely related to *Enterobacter iro* than to other *K*. *pneumoniae iro* in terms of both structure (including the *iroE* gene; see Fig. 1b, Additional file 14) and sequence (Additional file 10), suggesting it has been transferred from *Enterobacter* into a *K*. *pneumoniae* IncFIB_K_/FII_K_ plasmid backbone. pINF078-VP harboured multiple tandem copies of a 17,129 bp region containing *iroBCDEN* and 12 other genes of unknown function (Additional file 14). Long-read sequences (up to 70 kbp) spanning the non-repeat and repeat region of pINF078-VP confirmed at least *n* = 3 copies of the 17 kbp repeated sequence, whose mean read depth in the Illumina sequence data was 13.3 times that of the rest of the plasmid sequence, suggesting approximately 13 tandem copies.

### *Iuc*/*iro* lineage 5 loci are associated with plasmids originating from *E*. *coli*

Four *K*. *pneumoniae* isolates carried the *E*. *coli* variant *iuc5*; two of these also carried the *E*. *coli* variant *iro5* (see species trees in Additional file 10). Three *iuc5*+ isolates (including one with *iro5*) belonged to the globally disseminated, carbapenemase-producing *K*. *pneumoniae* CG258 (ST258, KPC+; ST437, KPC+; ST833, KPC−) and carried several AMR genes. Unfortunately, all four *iuc5*+ genomes were sourced from public databases and were available in draft form only, and the complete plasmid sequences could not be resolved. However, the *iuc5*+ contig sequences from *K*. *pneumoniae* share close homology with *iuc5*+*iro5*+ IncFII conjugative plasmids from *E*. *coli* that also carry AMR genes (e.g. p3PCN033, CP006635.1; D3 plasmid A, CP010141.1). Notably, all *iuc5* contigs from *K*. *pneumoniae* shared > 75% coverage and 98.19–99.95% identity to the p3PCN033 reference plasmid.

## Discussion

This study reveals significant genetic diversity underlying the biosynthesis of aerobactin and salmochelin in *K*. *pneumoniae* but shows the distribution of *iuc* and *iro* locus variants is highly structured within the population. Our data indicate that most of the burden of these hypervirulence-associated siderophores in the *K*. *pneumoniae* population is associated with two dominant virulence plasmids, which we define here as KpVP-1 and KpVP-2, that differ in terms of gene content (Fig. 2) and are each associated with co-segregating sequences of the non-self-transmissible IncFIB_K_ replicon, *iuc* and *iro* loci (Figs. 1 and 3). These dominant virulence plasmid types are each represented by one of the previously characterised *K*. *pneumoniae* virulence plasmids [18, 20], pK2044 (KpVP-1, encoding *iro1* and *iuc1*) and Kp152.145pII (KpVP-2, encoding *iro2* and *iuc2*); both also carry hypermucoidy determinants, and together, they account for 74% and 14% of the *iuc*+*iro*+ *K*. *pneumoniae* genomes analysed. Importantly, our data indicate that each of these common virulence plasmid variants is maintained at high prevalence in a small number of known hypervirulent clones: KpVP-1 in CG23 (96%, including pK2044 [18]), CG86 (80%, including pLVPK [19]) and CG65 (79%); KpVP-2 in CG66 (100%, including Kp152.145pII) and CG380 (100%) (Fig. 5). This suggests that both plasmid types can persist for long periods within a host bacterial lineage as it undergoes clonal expansion; indeed, our recent study of the evolutionary history of CG23 indicates that KpVP-1 has been maintained in this clonally expanding lineage for at least a century [22]. The lack of conjugation machinery is likely an important variable contributing to clonal expansion being the primary mode of dispersal over horizontal gene trasfer, although notably, we also detected KpVP-1 at low prevalence in numerous other *K*. *pneumoniae* lineages and KpVP-2 at low prevalence in one other lineage, suggesting the possibility of wider dissemination of both plasmid types by occasional transfer to new lineages (Fig. 5). Given the stability of the plasmids observed in several clonal groups, we speculate that some of these transfer events will result in the emergence of novel hypervirulent strains that can stably maintain the plasmid into the future. In contrast, the non-plasmid form of *iro* (*iro3*, occasionally integrated into the chromosomes of *K*. *pneumoniae* via ICE*Kp1*) was found at low prevalence (< 0.5%) and included just 1 of the 79 ST23 isolates analysed (NTUH-K2044, in which ICE*Kp1* was first described), 1/1 ST5, 1/21 ST111 (13%), 1/2 ST198, 2/15 CG25, 2/2 ST493 and 5/5 ST60. Hence, while ICE*Kp1* is somewhat dispersed in the *K*. *pneumoniae* population, it shows little evidence of stability within lineages, consistent with our previous observations regarding ICE*Kp* in general [8].

We also detected several novel *iuc*+ or *iro*+ plasmid types, the most common being the group of *iuc2a* plasmids (21% of all *iuc*+ isolates) that were detected in respiratory isolates from CG3, CG82 and CG90 and mostly originated from historical collections [51]. Interestingly, these combine an *iuc* sequence closely related to that of KpVP-2 (Fig. 1) with an IncFIB_K_ replicon sequence very close to that of KpVP-1 (Fig. 3) and showed substantial mosaicism and gene content variation (Figs. 2 and 4). The *iuc3* lineage was also quite common amongst the novel plasmid types (5.3% of all *iuc*+ isolates) and associated with a variety of diverse plasmids, most of which carried the IncFII *tra-trb* conjugative transfer region and thus are likely self-transmissible (Figs. 2 and 4). It is notable that *iuc2a* and *iuc3* plasmids were not only relatively rare in the bacterial population but also showed less evidence of stable maintenance within *K*. *pneumoniae* lineages (Fig. 5) and lower stability of gene content (Fig. 2) than the dominant KpVP-1 and KpVP-2 plasmids (Fig. 4). The position of *iuc2a* and *iuc3* in the *iuc* trees (Fig. 1, Additional file 10) suggests that both are derive from other *K*. *pneumoniae* loci; hence, we speculate it is the properties of the plasmids mobilising these loci, and not the siderophore biosynthesis loci themselves, that makes these variants less widespread in the *K*. *pneumoniae* population. This variation in gene content may be a consequence of self-transmissibility, exposing the plasmids to a wider gene pool of host bacteria and providing opportunities for gene content diversification, which could potentially include AMR genes. Notably, the *iuc3* plasmids carry an arsenal of additional virulence loci involved in iron metabolism and resistance to heavy metals, reminiscent of KpVP-1 (Fig. 2).

The other novel plasmids appear to derive from outside *K*. *pneumoniae* (Fig. 1, Additional file 10). Most concerning are the four *E*. *coli*-derived plasmids we detected carrying *iuc5* (and occasionally *iro5*) in the USA and Brazil, three of which were found in the MDR hospital outbreak-associated clone CG258. Whether these aerobactin plasmids harbour AMR genes as they do in *E*. *coli* is not currently resolvable; however, it seems that conjugative *E*. *coli* plasmids such as D3 plasmid A do have the potential to deliver hypervirulence and multidrug resistance to *K*. *pneumoniae* strains in a single step. A recent study of *K*. *pneumoniae* submitted to Public Health England used PCR to screen for isolates carrying both carbapenemase genes and *rmpA*, as a marker of the virulence plasmid, and identified a plasmid harbouring *iuc*, *rmpA*, *rmpA2* and the AMR genes *sul1*, *sul2*, *armA*, *dfrA5*, *mph(A)* and *aph(3′)-VIb* [28]. To our knowledge, this is the first report of a complete sequence of a *K*. *pneumoniae* plasmid harbouring both AMR and virulence genes. The isolate (ST147) was not included in our original screen; however, subsequent analysis using *Kleborate* plus manual inspection of the plasmid sequence reveals it carries *iuc1* (AbST63, a novel single locus variant of AbST1 which is typical of hypervirulent clones CG23, CG65 and CG86) and appears to be a mosaic carrying sequences from KpVP-1 (40% coverage), an IncFII *tra-trb* conjugative transfer region and transposons carrying AMR genes.

The presence of aerobactin synthesis loci in the *iuc5*+ *K*. *pneumoniae* isolates we identified here was not reported in the original studies [52, 53], and thus, it is not known whether they actually produce aerobactin or show enhanced virulence. This highlights the need to raise awareness of the *iuc* and *iro* loci as potentially clinically relevant hypervirulence factors and to screen for them in isolates and genome data. The latter, we aim to facilitate via the genotyping schemes established here, which can be used to easily screen new genome assemblies using Kleborate (https://github.com/katholt/Kleborate/) or BIGSdb-*Kp* (http://bigsdb.pasteur.fr/klebsiella/klebsiella.html), or new short-read data sets using SRST2 (https://github.com/katholt/srst2). PCR primers suitable for screening for *iro* and *iuc* can be found in Lee et al. [54]. Notably, many studies rely on the hypermucoidy phenotype to identify hypervirulent strains; however, this is dependent on growth conditions [55], and recent studies indicate that aerobactin synthesis is a more important virulence determinant [13, 14, 16]. Our data suggest that hypermucoidy screening would typically pick up most of the common aerobactin plasmids KpVP-1, KpVP-2 and *iuc2a*+ plasmids, but not those carrying *iuc3* or the *iuc5* plasmids from *E*. *coli*. Additionally, it is important not to conflate the presence of the core chromosomal receptor gene *iutA* with the ability to synthesise aerobactin, which is encoded in the *iuc* locus [6]. False-positive detection of the aerobactin locus version of *iutA* can be avoided by using an identity threshold of < 20% divergence. Tellurite resistance has also been suggested as a phenotypic screen to identify hypervirulent isolates of CG23, CG65 and CG86 [56]; our data confirm this is a good marker for KpVP-1 (92.6% carry *ter*) but not for other aerobactin plasmid types (Additional file 12).

## Conclusions

Our results illuminate that distinct virulence plasmid variants are associated with the various hypervirulent *K*. *pneumoniae* lineages but also highlight that these alongside other plasmids and MGEs can shuttle aerobactin and salmochelin synthesis loci to other lineages, threatening the emergence of novel hypervirulent strains. Indeed, reports of MDR clones acquiring *iuc* plasmids appear to be increasing in incidence, particularly in China [27, 29, 57–59] and have been associated with increased morbidity and mortality. The AbST and SmST typing schemes developed in this study provide an important resource to identify and monitor the movement of *iro* and *iuc* loci and associated MGEs in *K*. *pneumoniae* genomes; which will be important to detect and contain these emerging threats. Genotyping with our tools reveals the *iuc* plasmid identified in the recently reported fatal hospital outbreak of carbapenemase-producing ST11 in Beijing is a variant of KpVP-1 that carries *iuc1* (AbST1) and a single copy of *rmpA* but lacks the *iro* locus [27]. In this strain, the aerobactin plasmid does not carry any AMR determinants; the carbapenemase gene *bla*_KPC_ and several other AMR genes were located on other plasmids. Concerningly, the ability for the virulence plasmids to be maintained in *K*. *pneumoniae* lineages suggests that once established in the MDR hospital outbreak-associated clones, they may become quite stable. The initial report of *iuc*+ KPC+ ST11 in China prompted multiple other groups to report the detection of the same strain in their hospitals [60–62], suggesting this strain may indeed be emerging as a persistently hypervirulent and MDR form of *K*. *pneumoniae*. Genomic surveillance and control of the spread of such ‘dual-risk’ strains, or indeed even plasmids combining both characteristics of MDR and hypervirulence, clearly needs to be reinforced; the present work will bolster efforts to understand and limit the emergence of infections caused by *K*. *pneumoniae* strains carrying the high virulence determinants aerobactin and salmochelin.

## Additional files

Additional file 1: Strain information for genomes included in this study. (XLS 426 kb)

Additional file 2: General features of reference plasmids or incomplete plasmid sequences carrying *iro* and/or *iuc*. (DOC 48 kb)

Additional file 3: Summary of replicon sequences from isolates INF151, INF237 and INF078. (DOC 39 kb)

Additional file 4: Aerobactin sequence types (AbSTs) and corresponding alleles. (TXT 2 kb)

Additional file 5: Salmochelin sequence types (SmSTs) and corresponding alleles. (TXT 1 kb)

Additional file 6: Representative Enterobacterales genome sequences included in *iro* and *iuc* phylogenetic analysis. (CSV 6 kb)

Additional file 7: Phylogenetic relationships between the predicted amino acid sequences encoded by aerobactin (*iuc*) and salmochelin (*iro*) locus sequence types. Each tip represents a translated amino acid sequence for an aerobactin sequence type (AbST, in a) or salmochelin sequence type (SmST, in b). Lineages defined from nucleotide sequences (see tree in Fig. 1) are highlighted and labelled. (PDF 135 kb)

Additional file 8: Single nucleotide variants and nucleotide divergence (%) observed within (shaded in grey) and between the aerobactin-encoding *iuc* lineages. (DOC 33 kb)

Additional file 9: Single nucleotide variants and nucleotide divergence (%) observed within (shaded in grey) and between the salmochelin-encoding *iro* lineages. (DOC 30 kb)

Additional file 10: Phylogenetic trees for salmochelin and aerobactin encoding *iuc* locus in *K*. *pneumoniae* and other Enterobacterales bacteria. Trees represent show a midpoint-rooted maximum likelihood phylogeny for representative sequences identified in various Enterobacterales species (listed in Additional file 6). Tip colours indicate the genetic context of the locus: black = plasmid, red = chromosome. *K*. *pneumoniae iro* lineages defined in Fig. 1 are coloured; other species-specific clades are highlighted in grey; individually labelled tips within highlighted clades indicate exceptions to the species label of the clade. Salmochelin trees were inferred using the *iroB* gene alone (panel a), which show a highly divergent form in *Salmonella*. Panel (b) shows a tree inferred from all four genes of the typical *K*. *pneumoniae iro* locus (*iroBCDN*), excluding the distantly related *Salmonella* variant, to increase resolution within the group containing *Klebsiella*. Similarly, aerobactin trees were inferred using the *iucB* gene alone (panel c) to show the overall structure, and separately for the full set of genes in the *K*. *pneumoniae* locus (*iucABCD*, *iutA*) to provide greater resolution within the group containing *Klebsiella* (panel d). (PDF 297 kb)

Additional file 11: Summary of aerobactin-encoding iuc and salmochelin-encoding *iro* loci BLAST hit. (CSV 1 kb)

Additional file 12: Prevalence of virulence loci and plasmid replication loci amongst isolates with virulence plasmids. (CSV 1 kb)

Additional file 13: Conservation of coding sequences from KpVP-2 and *iuc2a*+ reference plasmids amongst isolates carrying plasmid-encoded *iuc2* or *iuc2a* loci. Cells show circularised heatmaps indicating the frequency of each gene in a given reference plasmid (column), amongst isolates of a given chromosomal sequence type (ST) or clonal group (CG) (rows) that carry either *iuc2* (CG66, CG380) or *iuc2a* (others). Around each circle, genes are ordered by their order in the corresponding reference plasmid. (PDF 2864 kb)

Additional file 14: Genetic structure of 17 kbp repeat region in plasmid pINF078-VP and the chromosomally-encoded *E*. *cloacae iro* region. Shaded area indicates a homologous region of 95% nucleotide identity shared between the two sequences. Coding sequences are represented by the arrows and coloured according to the closest Enterobacteriaceae species match as indicated in the legend. (PDF 172 kb)

## Abbreviations

AbST: Aerobactin sequence type
AMR: Antimicrobial resistance
CA: Community-associated
CG: Clonal group
CI: Confidence interval
Ent: Enterobactin
HA: Healthcare-associated
HGT: Horizontal gene transfer
ICEs: Integrative and conjugative elements
Iro: Salmochelin
Iuc: Aerobactin
MDR: Multidrug-resistant
MGEs: Mobile genetic elements
MLST: Multi-locus sequence typing
OR: Odds ratio
SmST: Salmochelin sequence type
ST: Sequence type
Ybt: Yersiniabactin
