# Supplementary material for: Tracking key virulence loci encoding aerobactin and salmochelin siderophore synthesis in Klebsiella pneumoniae
Source: Genome Med. 2018 Oct 29;10:77. doi: 10.1186/s13073-018-0587-5 (PMC6205773; doi:10.1186/s13073-018-0587-5)
Supplement: Supplementary file 2 — General features of reference plasmids or incomplete plasmid sequences carrying iro and/or iuc. (DOC 48 kb) [file 13073_2018_587_MOESM2_ESM.doc]

**Additional file 2. General features of reference plasmids or incomplete plasmid sequences carrying *iro* and/or *iuc*.**

| **Lineage** | **Reference Strain** | **Contig/plasmid size** | **Plasmid rep(s)** | **Additional AMR/virulence loci** | **Comments** | **Accession** |
| --- | --- | --- | --- | --- | --- | --- |
| *iuc1*  *iro1* | pK2044 | 224.2 kb (complete) | IncFIB_K_, IncHI1B | VIR: *rmpA, rmpA2, fec* iron acquisition, *pbr-pco* copper resistance, *sil* silver resistance, *terXYW* and *terZABCDEF* tellurite resistance, *vagCD* | KPVP-1  Most common virulence plasmid | AP006726.1 |
| *iuc2*  *iro2* | Kp52.145 plasmid II | 121.7 kb (complete) | IncFIB_K_ | VIR: *rmpA, fec* iron acquisition | KPVP-2  Second most common virulence plasmid | FO834905.1 |
| *iuc2A* | pINF151_01-VP | 138.1 kb (complete) | IncFIB_K_ | VIR: iron acquisition locus from *Serratia marcescens*, *fur*, TonB receptor, *rmpA* |  | This study. Accession QWFT01000004. |
|  | pINF237_01-VP | 133.7 kb (complete) | IncFIB_K_ | VIR: iron acquisition locus from *Serratia marcescens, fec* iron acquisition, *fur*, TonB receptor, *rmpA* |  | This study. Accession CP032834. |
|  | NCTC12463 | 263.5 kb (contig) | IncFIB_K_, IncFII(pCoo), IncFIA | VIR: *fec* iron acquisition, *terXYQ* and *terZABCDEF* tellurite resistance |  | GCA_900451415.1 |
|  | NCTC9635 | 230.6 kb (Pacbio contig, trimmed) | IncFIB_K_, IncFIA | VIR: iron acquisition locus from *Serratia marcescens, fec* iron acquisition, *rmpA, iroB, terXYQ* and *terZABCDEF* tellurite resistance, cloacin |  | NCTC 3000 project* |
|  | NCTC8895 | 193.7 kb (Pacbio contig, trimmed) | IncFIB_K_, IncFIA | VIR: iron acquisition locus from *Serratia marcescens, fec* iron acquisition, *fur* (2 copies), TonB receptor, *rmpA*, cloacin |  | NCTC 3000 project* |
|  | NCTC9662 | 96.5 kb (Pacbio contig, trimmed) | IncFIB_K_ | VIR: *fur,* partial *iro* locus |  | NCTC 3000 project* |
| *iuc3* | NCTC11676 | 189.8 kb (Pacbio contig, trimmed) | IncFII(*Y. pestis*), IncFIB(*E. coli*), IncFIB(*E. asburiae*) | VIR: colicin E3+immunity, fimbrial biogenesis, TonB receptor, *fec* iron acquisition, *fur* |  | NCTC 3000 project* |
|  | NCTC11697 | 155.4 kb (contig) | IncFII, IncFIB_K_ | VIR: fimbrial protein, *fec* iron acquisition, *fur*, cloacin |  | NCTC 3000 project* |
| *iro4* | pINF078_VP | 399.9 kb (complete) | IncFII, IncFIB_K_ | VIR: 13 copies of *iro,* TonB receptor | *iro* most closely related to *E. cloacae* variant of *iro* | This study. Accession CP032832. |
| *iuc5*  (+*iro5)* | p3PCN033 | 161.5 kb (complete) | IncFIC(FII), IncFIB | AMR: *cat* (2 copies)*, aph3-Ia, strAB, sulII, etsABC* macrolide efflux, *oqxAB, dfrA*, *bla_TEM_, tetDCE*  VIR: *mer* mercury resistance, *sitABCD* iron acquisition, *hlyF* hemolysin F, colicin | *iro* most closely related to *E. coli* variant of *iro* | CP006635.1 |

*NCTC 3000 Project. Genome sequences available at https://www.sanger.ac.uk/resources/downloads/bacteria/nctc/
