# Supplementary material for: Tracking key virulence loci encoding aerobactin and salmochelin siderophore synthesis in Klebsiella pneumoniae
Source: Genome Med. 2018 Oct 29;10:77. doi: 10.1186/s13073-018-0587-5 (PMC6205773; doi:10.1186/s13073-018-0587-5)
Supplement: Supplementary file 3 — Summary of replicon sequences from isolates INF151, INF237 and INF078. (DOC 39 kb) [file 13073_2018_587_MOESM3_ESM.doc]

**Additional file 3. Summary of replicon sequences from strains INF151, INF237 and INF078**

|  | **Replicon** | **Length (bp)** | **Comments** | **Accession** |
| --- | --- | --- | --- | --- |
| INF151 | Chromosome | unresolved |  | QWFT01000001- QWFT01000003 |
|  | pINF151_01-VP | 138,147 |  | QWFT01000004 |
|  | pINF151_02 | 83,146 |  | QWFT01000005 |
|  | pINF151_03 | 75,803 |  | QWFT01000006 |
|  | pINF151_04 | 65,865 |  | QWFT01000007 |
|  | pINF151_05 | 8,535 |  | QWFT01000008 |
|  | pINF151_06 | 6,829 |  | QWFT01000009 |
| INF237 | Chromosome | 5,203,185 |  | CP032833 |
|  | pINF237_01-VP | 133,713 |  | CP032834 |
|  | pINF237_02 | 93,621 |  | CP032835 |
|  | pINF237_03 | 76,772 |  | CP032836 |
|  | pINF237_04 | 44,993 |  | CP032837 |
|  | pINF237_05 | 6,704 |  | CP032838 |
| INF078 | Chromosome | 5,295,441 |  | CP032831 |
|  | pINF078-VP | 399,913 | 13 copies of *iro* | CP032832 |
