# Supplementary figures and images for: Tracking key virulence loci encoding aerobactin and salmochelin siderophore synthesis in Klebsiella pneumoniae
Source: Genome Med. 2018 Oct 29;10:77. doi: 10.1186/s13073-018-0587-5 (PMC6205773; doi:10.1186/s13073-018-0587-5)

**A**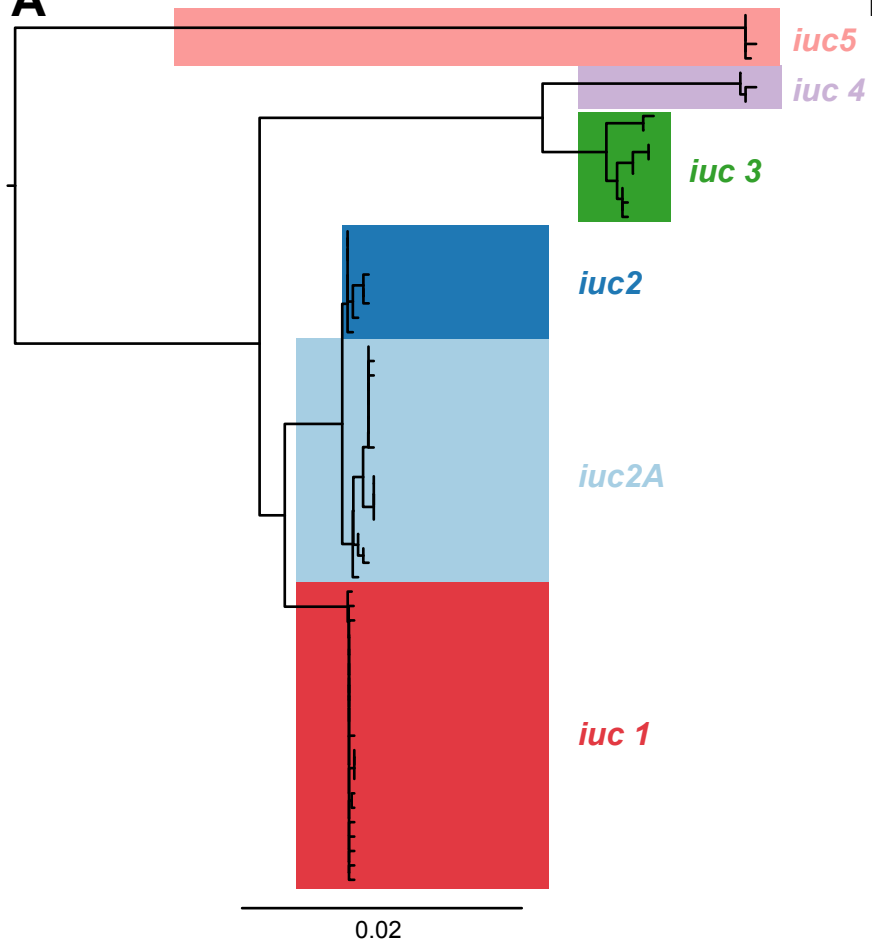**B**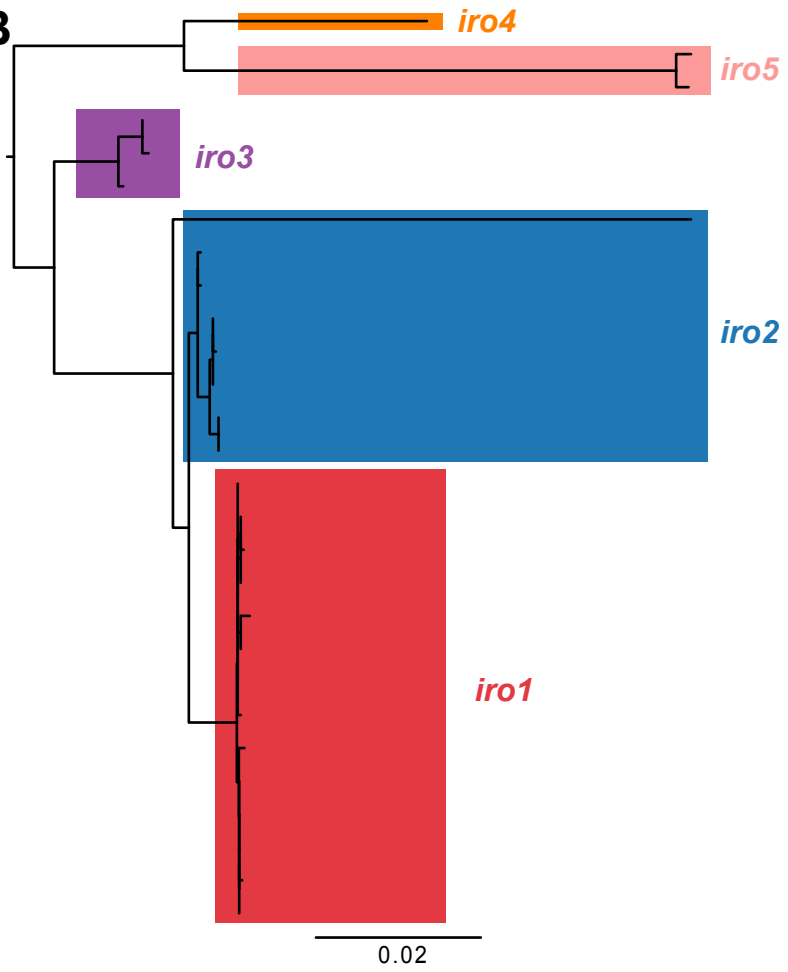

Supplement: Supplementary file 7 — Phylogenetic relationships between the predicted amino acid sequences encoded by aerobactin (iuc) and salmochelin (iro) locus sequence types. Each tip represents a translated amino acid sequence for an aerobactin sequence type (AbST, in a) or salmochelin sequence type (SmST, in b). Lineages defined from nucleotide sequences (see tree in Fig. 1) are highlighted and labelled. (PDF 135 kb) [file 13073_2018_587_MOESM7_ESM.pdf]

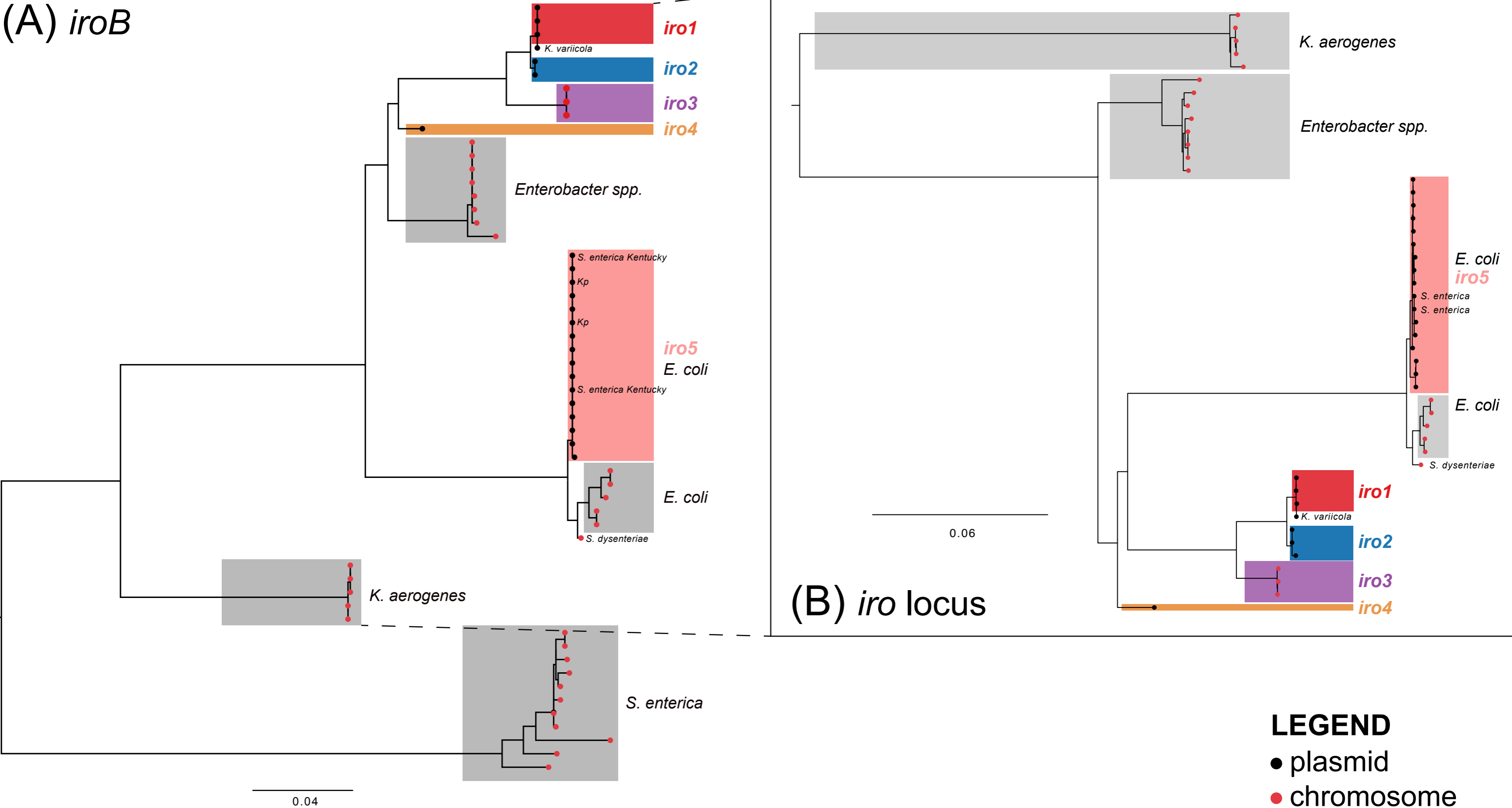

**LEGEND**

- plasmid
- chromosome

Supplement: Supplementary file 10 — Phylogenetic trees for salmochelin and aerobactin encoding iuc locus in K. pneumoniae and other Enterobacterales bacteria. Trees represent show a midpoint-rooted maximum likelihood phylogeny for representative sequences identified in various Enterobacterales species (listed in Additional file 6). Tip colours indicate the genetic context of the locus: black = plasmid, red = chromosome. K. pneumoniae iro lineages defined in Fig. 1 are coloured; other species-specific clades are highlighted in grey; individually labelled tips within highlighted clades indicate exceptions to the species label of the clade. Salmochelin trees were inferred using the iroB gene alone (panel a), which show a highly divergent form in Salmonella. Panel (b) shows a tree inferred from all four genes of the typical K. pneumoniae iro locus (iroBCDN), excluding the distantly related Salmonella variant, to increase resolution within the group containing Klebsiella. Similarly, aerobactin trees were inferred using the iucB gene alone (panel c) to show the overall structure, and separately for the full set of genes in the K. pneumoniae locus (iucABCD, iutA) to provide greater resolution within the group containing Klebsiella (panel d). (PDF 297 kb) [file 13073_2018_587_MOESM10_ESM.pdf]

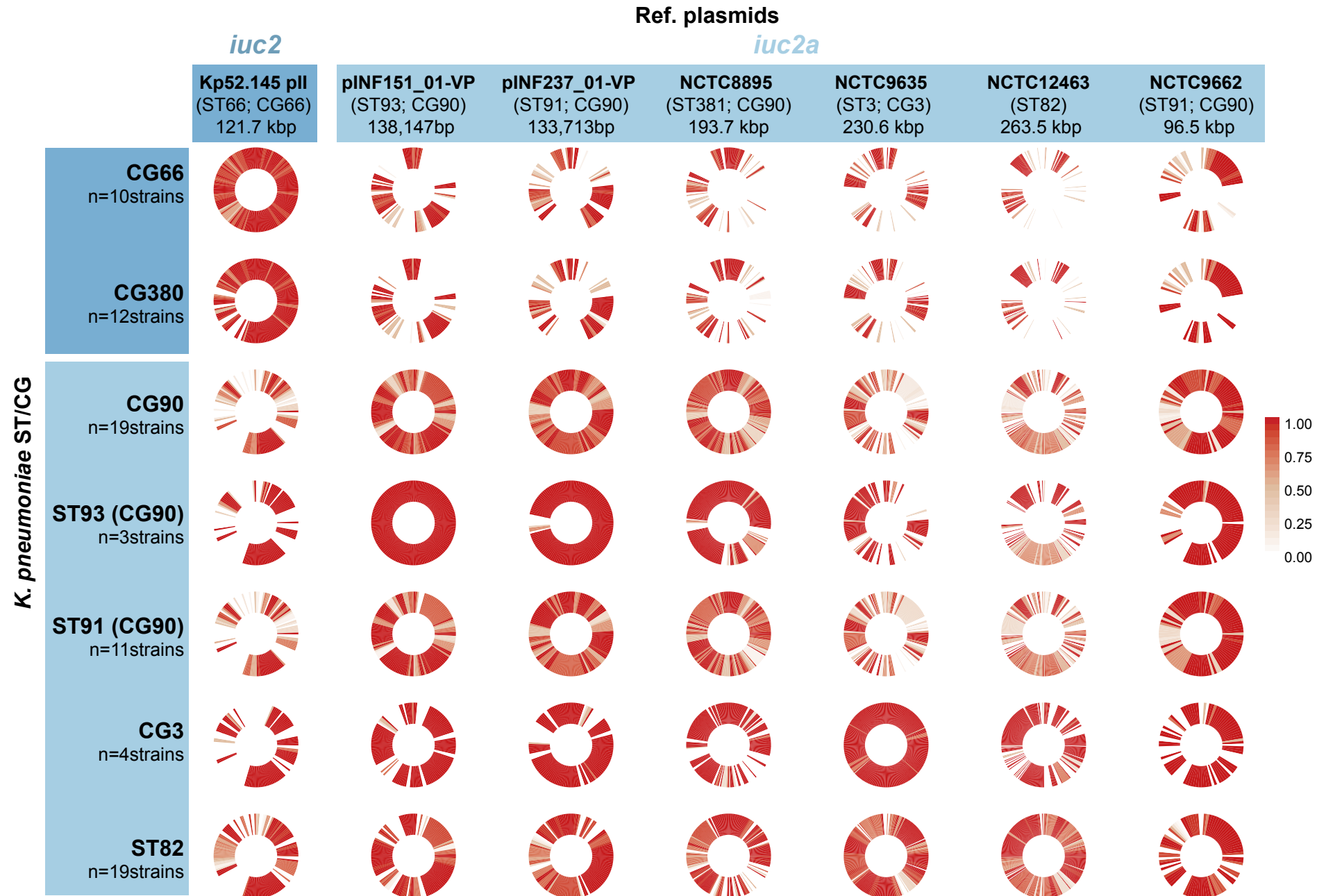

Supplement: Supplementary file 13 — Conservation of coding sequences from KpVP-2 and iuc2a+ reference plasmids amongst isolates carrying plasmid-encoded iuc2 or iuc2a loci. Cells show circularised heatmaps indicating the frequency of each gene in a given reference plasmid (column), amongst isolates of a given chromosomal sequence type (ST) or clonal group (CG) (rows) that carry either iuc2 (CG66, CG380) or iuc2a (others). Around each circle, genes are ordered by their order in the corresponding reference plasmid. (PDF 2864 kb) [file 13073_2018_587_MOESM13_ESM.pdf]
