# Supplementary material for: Tracking key virulence loci encoding aerobactin and salmochelin siderophore synthesis in Klebsiella pneumoniae
Source: Genome Med. 2018 Oct 29;10:77. doi: 10.1186/s13073-018-0587-5 (PMC6205773; doi:10.1186/s13073-018-0587-5)
Supplement: Supplementary file 8 — Single nucleotide variants and nucleotide divergence (%) observed within (shaded in grey) and between the aerobactin-encoding iuc lineages. (DOC 33 kb) [file 13073_2018_587_MOESM8_ESM.doc]

**Additional file 8. Single nucleotide variants and nucleotide divergence (%) observed within (shaded in grey) and between the aerobactin-encoding *iuc*** lineages.

|  | *iuc1* | *iuc2* | *iuc2a* | *iuc3* | *iuc4* | *iuc5* |
| --- | --- | --- | --- | --- | --- | --- |
| *iuc1* | 1-9 SNPs  0.013-0.113 | 87-96 SNPs  1.091-1.204 | 85-99 SNPs  1.066-1.242 | 308-324 SNPs  3.863-4.063 | 339-348 SNPs  4.251-4.364 | 708-714 SNPs  8.879-8.954 |
| *iuc2* |  | 1-17 SNPs  0.013-0.213 | 1-21 SNPs  0.013-0.263 | 278-290 SNPs  3.486-3.637 | 354-360 SNPs  4.439-4.515 | 717-724 SNPs  8.992-9.080 |
| *iuc2a* |  |  | 1-17 SNPs  0.013-0.213 | 276-293 SNPs  3.461-3.674 | 352-363 SNPs  4.414-4.552 | 716-723 SNPs  8.979-9.067 |
| *iuc3* |  |  |  | 1-40 SNPs  0.013-0.502 | 164-177 SNPs  2.057-2.220 | 901-916 SNPs  11.299-11.487 |
| *iuc4* |  |  |  |  | 1-3 SNPs  0.013-0.038 | 951-956 SNPs  11.926-11.989 |
| *iuc5* |  |  |  |  |  | 1-4 SNPs  0.013-0.050 |
