# Supplementary material for: Tracking key virulence loci encoding aerobactin and salmochelin siderophore synthesis in Klebsiella pneumoniae
Source: Genome Med. 2018 Oct 29;10:77. doi: 10.1186/s13073-018-0587-5 (PMC6205773; doi:10.1186/s13073-018-0587-5)
Supplement: Supplementary file 9 — Single nucleotide variants and nucleotide divergence (%) observed within (shaded in grey) and between the salmochelin-encoding iro lineages. (DOC 30 kb) [file 13073_2018_587_MOESM9_ESM.doc]

**Additional file 9. Single nucleotide variants and nucleotide divergence (%) observed within (shaded in grey) and between the salmochelin-encoding *iro*** lineages.

|  | *iro1* | *iro2* | *iro3* | *iro4* | *iro5* |
| --- | --- | --- | --- | --- | --- |
| *iro1* | 1-8 SNPs  0.001-0.399 | 40-57 SNPs  0.483-0.689 | 224-259 SNPs  2.707-3.130 | 513-519 SNPs  6.199-6.272 | 927-934 SNPs  11.202-11.287 |
| *iro2* |  | 1-30 SNPs  0.001-0.363 | 219-254 SNPs  2.647-3.069 | 503-511 SNPs  6.079-6.175 | 918-924 SNPs  11.094-11.166 |
| *iro3* |  |  | 1-33 SNPs  0.001-0.399 | 457-466 SNPs  5.523-5.631 | 899-907 SNPs  10.864-10.961 |
| *iro4* |  |  |  | NA | 711-713 SNPs  8.592-8.616 |
| *iro5* |  |  |  |  | 25 SNPs  0.302 |

Note. *iro4* was observed in a single genome.
