# Supplementary material for: Tracking key virulence loci encoding aerobactin and salmochelin siderophore synthesis in Klebsiella pneumoniae
Source: Genome Med. 2018 Oct 29;10:77. doi: 10.1186/s13073-018-0587-5 (PMC6205773; doi:10.1186/s13073-018-0587-5)
Supplement: Supplementary file 14 — Genetic structure of 17 kbp repeat region in plasmid pINF078-VP and the chromosomally-encoded E. cloacae iro region. Shaded area indicates a homologous region of 95% nucleotide identity shared between the two sequences. Coding sequences are represented by the arrows and coloured according to the closest Enterobacteriaceae species match as indicated in the legend. (PDF 172 kb) [file 13073_2018_587_MOESM14_ESM.pdf]

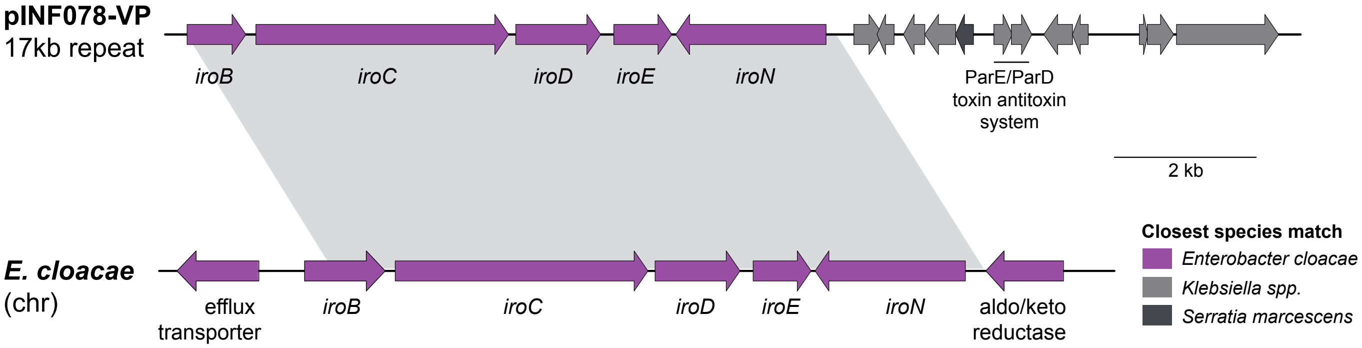
pINF078-VP
17kb repeat
iroB
iroC
iroD
iroE
iroN
ParE/ParD
toxin antitoxin
system
2 kb
E. cloacae
(chr)
efflux
transporter
iroB
iroC
iroD
iroE
iroN
aldo/keto
reductase
Closest species match
Enterobacter cloacae
Klebsiella spp.
Serratia marcescens
